# Sex, Bugs and Rock ‘n’ Roll: Insects in Music Videos

**DOI:** 10.3390/insects12070616

**Published:** 2021-07-07

**Authors:** Joseph R. Coelho

**Affiliations:** Biology Program, The Institute for Franciscan Environmental Studies, Quincy University, 1800 College Avenue, Quincy, IL 62301, USA; j.coelho42@quincy.edu; Tel.: +1-217-228-5432 (ext. 3268)

**Keywords:** insect, music, video, Lepidoptera, Hymenoptera, animation, chimera

## Abstract

**Simple Summary:**

Music videos were examined on YouTube for insect content. The types of insects shown, the year of issue, and themes were recorded. The most common insects seen in music videos were butterflies and moths. Bees, ants, and wasps were the second most common, while beetles, spiders, flies, and grasshoppers/crickets were tied for third place. Insect music videos are becoming increasingly common, perhaps because the total number of videos being issued is also increasing. Large numbers of insects were common in about one quarter of the videos, while insects with human features were in about one fifth. Giant insects were seen in only one twentieth of the videos. Many insect music videos and their associated songs have been very successful, with ten number one songs and four music video awards. Animation of various types was used in many insect videos, while live footage and photographs allowed identification of many of the insects. The types of insects shown, the themes represented and the success of insect music videos seem to indicate that human attitudes toward insects are trending toward more positive values, especially relative to those in early horror films, which were exclusively designed to convey horror.

**Abstract:**

The appearance of insects in music videos was examined. The most common taxa observed were Lepidoptera, then Hymenoptera, with Coleoptera, Araneae, Diptera, and Orthoptera essentially tied for third most represented. Insect music videos have increased in frequency over time, probably as an artifact of industry growth. Swarms and infestations were common in insect videos (appearing in 26%), as were chimeric insectoid humans (19%), and, to a lesser extent, giant insects (5%), but not all of these representations were used to induce horror. Some insect music videos have garnered awards, and many of the songs associated with them have been very successful. There were many animated insect sequences, but also images of specimens that were sufficiently detailed to allow identification of the species. The insect groups observed reflect both positive and negative values. There is some indication that insects are not viewed in such a negative light as they once were, providing hope for improving attitudes of humans toward insects.

## 1. Introduction

Human perceptions of and attitudes toward insects have been examined via the appearance of insects in a large variety of media, including art, literature, and music. An early review of cultural entomology [1] examined the state of the discipline broadly. A recent review [2] suggests that insects are viewed primarily negatively. However, studies have yielded variable results. Popular music that uses insects as cover art primarily uses the aesthetically pleasing Lepidoptera [3], whereas musical artists, albums and song titles favor the Hymenoptera, which carries both positive and negative connotations [4,5,6].

Given the recent recognition of the global decline in insect populations [7], it is critical that we understand how humans perceive insects and perhaps how we leverage that knowledge to conserve insects [8]. A multipronged approach examining the concept from diverse perspectives is likely to lead to a broad understanding. Media that are relatively new and appealing to youth may have a greater impact than older forms. The music video, though appearing sparingly in a variety of venues for decades previously, seemed to burst onto the scene in the United States of America on 1 August 1981, when the cable television channel MTV launched. From the beginning, the channel was steeped in insectile offerings. The first video shown was “Video Killed the Radio Star” by The Buggles, the band’s name being an insect pun. One of the early important acts was Adam and the Ants. Although MTV’s programming is now more diverse, the music video changed the music industry and American popular culture. The music video, as a brief bite of images and sound, is the perfect form for the modern short attention span. It entertains the viewer while promoting the artist and their music. Hence, the imagery used in it is expected to be carefully chosen. This study examines the use of insects in music video, a relatively recent popular medium, on the premise that the insects that appear and the manner in which they are used will reflect current human perceptions and attitudes toward insects.

## 2. Materials and Methods

The appendices from previous works [3,4,9] were used to search YouTube for videos featuring any of the artists, albums, or songs listed. An additional list of 164 albums featuring insect cover art compiled by the author was similarly used. Internet searches for lists and collections of insect or animal songs also yielded a few videos. 

Most of the songs were, in fact, found on YouTube, but the majority found in this way did not demonstrate insects at any point. Many videos featured a static image of the album cover (often featuring an insect), but since cover art has already been examined [3], such videos were not collected as data. If the video was not a static image (or a simple rotation of a few images) and it used insects or insectile creatures, then it was added to the data set. Videos utilizing insect sounds without insect imagery were not included. Videos that were derived from full-length motion pictures or television shows were excluded unless the artist released the video separately. No children’s or educational songs were included.

I also searched keywords from previous studies [3,4,9]. Searching YouTube produces a never-ending scroll of offerings, even for less common entomological terms. I examined approximately the first 200. Hovering the cursor over a video thumbnail provided a preview, allowing me to discern whether it was a static image or a motion video. Video settings allow one to vary the playback speed. Sometimes the video was speeded up to get through concert footage, etc. where no insects were expected. Sometimes the playback rate was slowed down or paused, and the image size maximized in order to aid in identification of animals shown very briefly.

Other insect music videos were discovered opportunistically, such as by watching television shows, or by soliciting members of social media sites, such as the Facebook page Cultural Entomology. These efforts collectively yielded a reasonable sample size, but it should still be considered a sample rather than a comprehensive census, as doubtless there are additional insect videos that were not discovered. Searches were conducted primarily during December 2020 and January 2021.

If a video contained an image or visual reference to an insect or arthropod (excluding crustaceans), the artist, album (if any), song title, and the year the song was released were recorded. Often other internet resources (such as Wikipedia) were accessed to complete these portions of the data. A link to the video, the taxa featured and a brief description of the insects and their role in the video were recorded. The appearance of swarms or infestations, chimeric insectoids, and giant insects was also recorded. Only official videos made by the artist, record company, or director were used. Fan videos are becoming common and may not represent the original artist’s intent. Raw data are available in the Supplementary Materials.

The taxonomy used follows Table 1, with arthropods classified primarily to Order, with a few exceptions. Though fleas are now members of Mecoptera [10], I labeled them Siphonaptera in order to be more specific because there were no scorpionflies in the data. For non-insect arthropods, I used the categories Araneae for spiders and tarantulas and Myriapoda for centipedes and millipedes. “Other Arachnida” was created as a category to lump together the few scorpions, tailless whipscorpions, vinegaroons, harvestmen, and ticks. Unidentifiable insects and insectoids were placed in the category “Unknown”.

### 2.1. Date

Where possible, the year the song was originally published was recorded, though that was not necessarily the year the video came out. While they are typically close, there were a few cases where videos were produced upon reissue of the song. 

### 2.2. Themes

Swarms and infestations were counted if four or more insects appeared onscreen at once. Chimeric insectoids were counted when insects appeared with human features, or, more often, when humans appeared with insect features such as wings. Giant insects were recorded when the insect represented was much larger than actual size, except human-sized chimerics were not counted as giant.

### 2.3. Success

An attempt was made to determine the degree of success of music videos featuring insects. The MTV Video Music Award listings on Wikipedia were examined for videos that were in the data. The success of songs upon which insect videos were based was also examined. As the relevant records, such as the Billboard Top 100, were unavailable (behind a paywall), I searched Wikipedia for songs that I believed were popular. The entry for the song often listed its level of achievement. Only the songs that reached number one on a chart were recorded.

## 3. Results

### 3.1. Taxa

**Figure 1:**
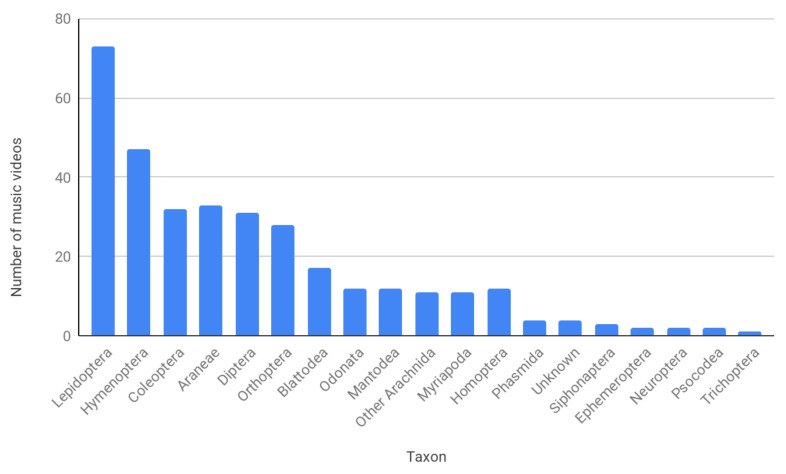
The distribution of arthropod taxa observed in music videos.

A total of 234 videos displaying insects were found. Lepidoptera were the most common, noted in 73 (31%) of these, and Hymenoptera, appearing in 47 (20%), were the second most common. Coleoptera, Araneae, Diptera, Orthoptera were essentially tied for third most common, at 12–14%. Other taxa were observed in less than 10% each of the total (Figure 1).

### 3.2. Date

**Figure 2:**
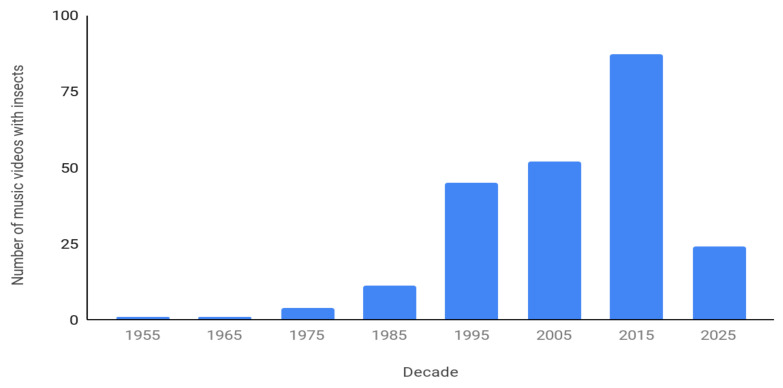
A histogram demonstrating the appearance of insects in music videos over time. The data are organized by decade from 1950 to 1959, and so on. The last bar only covers 2020 and part of 2021.

The most insect videos were produced recently, the largest decade being the 2010s (Figure 2). The numbers decrease as one goes back in time. The 2020s have a remarkable number already (24), and only one and a half years have passed. The oldest video listed is from 1955, although Artichoke’s “Bees”, released in 2010, uses footage from a 1951 documentary, *Bee City*.

### 3.3. Themes

**Figure 3:**
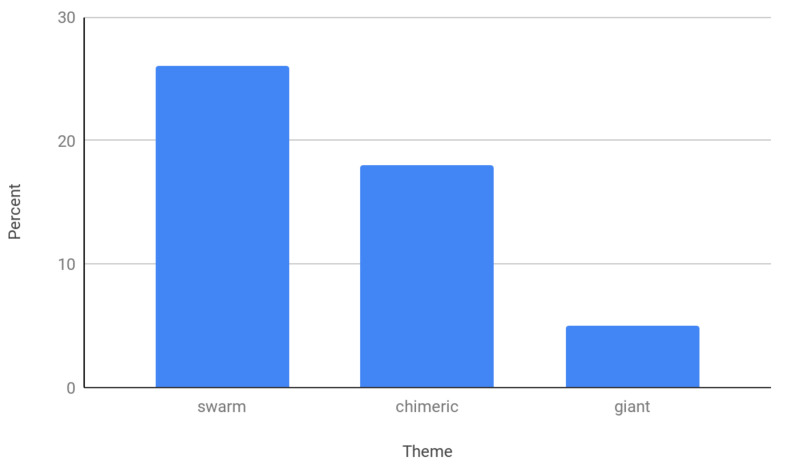
The relative appearance of three common themes in music videos: swarms, chimeric human/insect hybrids, and giant insects.

Swarms and infestations were common in insect videos, appearing in 60 (26%), while chimeric insectoids were also relatively abundant at 43 (19%). Giant insects were relatively scarce, appearing in only 12 (5%) videos (Figure 3).

Only 4% of the insect music videos were created by artists with insect names. Just 13% came from albums with insect names, but 50% of videos featuring insects had an insect reference in the title of the song.

### 3.4. Success

Of the 37 years in which MTV has issued Video Music Awards, videos featuring insects have won four, a greater than 10% winning rate (Table 2). 

Ten songs with videos that showed insects were identified that achieved number one status on one or more popularity charts (Table 3).

## 4. Discussion

### 4.1. Taxonomy

Lepidoptera being the most commonly insects featured in music videos is not terribly surprising, given that it is a visual medium. Butterflies and moths are the most abundant species in album covers [3], the second most common in rock music [4], and third in folk [5]. Butterflies are conspicuous and well-known to non-entomologists, appearing in artwork going back centuries. Their colorful wing patterns and elegant flight provide them with perhaps the greatest aesthetic value among insects. It probably helps that butterflies are relatively easy to animate, and live specimens are not difficult to obtain, such as the Painted Lady (*Vanessa cardui*) appearing in Maddie and Tae’s “Fly”.

Hymenoptera are the second most frequently observed insects in music videos. Bees compose the majority of these, followed by ants and wasps. This pattern probably follows the familiarity of most humans with this group. The popularity of hymenopterans may be attributable to their almost unique position of being able to represent both positive and negative traits, apparently leading to them being the most common insects in rock music album titles, song titles and artist names, as well as subjects of folk music [4,5,6]. Bees can suggest the sweetness of honey, ants thriftiness, hard work, and toughness, and wasps the threat of the sting. Bees’ perceived cuteness is utilized by frequent appearances of people in bee costumes, the best known being Blind Melon’s “No Rain”, in which an adorable little girl wears a bee costume throughout the entire video, and she is joined by bee-costumed adult dancers. The availability of live honeybees (*Apis mellifera*) likely facilitates their appearance in several videos, such as Missy Elliot’s “Work it”. The strength of ants is well demonstrated in Rammstein’s “Links 2 3 4”, in which a colony of ants, initially threatened by several large tiger beetles, rally their forces to wipe them out. The totemic quality of ants is represented by rap group Alien Ant Farm, which uses their presumably trademarked ant head logo in at least 6 videos. Wasps were almost always used in a terrorizing way. The recent appearance of Asian giant hornets (*Vespa mandarinia*) in North America has spawned at least two songs and videos, both with negative implications.

Araneae, Coleoptera, Diptera, and Orthoptera were approximately equally represented among music videos. Araneae are the most common arthropods in older feature films [11], perhaps indicating a shift in attitudes from negative to positive, though when shown in music videos, they were almost always used to inspire fear. Spiders and tarantulas are nearly all used to inspire horror, perhaps best illustrated by The Cure’s “Lullaby” in which spiderwebs and tarantulas are featured throughout, and the lead singer is swallowed by a spider in the end.

Beetles are often colorful enough to be co-opted for their aesthetic value, such as lightning bugs (Lampyridae), but the diversity of their uses defies classification, as scarabs may be used to represent death, while mealworms (*Tenebrio molitor* larvae) sometimes serve as stand-ins for maggots. These varied representations have resulted in a consistently moderate use of Coleoptera in music: beetles are the third most common group in cover art [3], fourth in folk songs [5], and seventh in rock songs [4]. 

Flies often inspire disgust, as do their larvae, maggots [12]. Dipterans are also well known to all and can be quite pestiferous. Hence, flies are the second most common in folk music [5], third in albums and songs (first in artist names) [4], and fourth in cover art [3]. In Alice in Chains’ “I Stay Away” (from the album *Jar of Flies*), flies are central to the story, and are presented in a fascinating mix of live specimens and claymation. Mosquitoes are also used with unpleasant implications. The Yeah Yeah Yeahs’ “Mosquito” features a hyper-realistic CGI mosquito taking a blood meal from a human hand for much of the duration of the video. Its abdomen swells to enormous proportions, and it gets squished in the end.

Orthoptera made a strong showing in insect videos, as they have in other media; they are 5th in folk music [5], 5th in rock artists and albums, and 6th in tracks [4]. This taxon gets a boost from the Moon Crickets, a rap group that sports their cricket logo in four different videos. Grasshoppers and crickets often represent favorable values [13] and could presumably be used to generate favorable feelings. Rather than being seen as a useful food source [13], swarms of locusts are used to generate fear in several videos. Machine Head’s “Locust” probably features the biggest swarm of any species and the greatest number of insects in any video, with dense clouds of migratory locusts contributing to the strong menacing effect of the music.

Cockroaches were the seventh most popular in videos, as well as in rock music tracks [4]. Nearly always, these are the go-to species for inspiring disgust. Cockroaches are the second most disgusting arthropods [12]. Familiar to all as household pests, swarms of cockroaches appear in the video for Papa Roach’s “Between Angels and Insects”.

Other taxa appeared in numbers too small to make any strong inferences, though most seemed to conform to stereotypical roles, such as fleas and lice being negative. Mantids and phasmids made a small showing, usually with live specimens. A few obscure insects did appear, including a dobsonfly, a lacewing, a mayfly and a brief glimpse of a caddisfly. This rarity is, again, consistent with most people’s ignorance of these species.

Less common non-insect arthropods were dominated by scorpions and myriapods, but also included two tailless whipscorpions, two ticks, two vinegaroons and a single harvestman. Scorpions and centipedes are among the most feared arthropods [12], which may account for their frequency among non-insect arthropods. 

Conspicuous by their absence were the Hemiptera (Heteroptera), which are common in rock music in part because of the use of the generic term “bug” and the appearance of bed bugs in many songs [4]. The term “bug” alone does not lend itself to illustration. No bed bugs appeared in any music videos, perhaps because they prove difficult to obtain. Homopterans in the form of cicadas did show up and were generally cast in a favorable light. In fact, a recent mass emergence of *Magicicada* was celebrated by Trasea’s “Cicada Serenade” (Brood X), Southern Culture on the Skids’ “Cicada Rock 2020 (Brood IX)”, and an earlier brood by Lloyd H. Miller with “Seventeen Years (a cicada love song)”.

Half of the music videos with insects in them also had insects in the title of the song. Most often, the insect named in the song title was at least one of those appearing in the video. As seen previously [4], insect themes can be correlated, as an artist with an insect name is likely to produce insect-related albums and songs. However, as artists with insect names were relatively rare in this study, only two completed the trifecta, having an insect video of an insect song from an insect album: Papa Roach’s “Between Angels and Insects” from *Infest* and Wu-Tang Killa Bees “Killa Beez” from *The Sting*.

### 4.2. Animation

Leskosky and Berenbaum [14] suggest that one of the reasons that insects are uncommon in early animated films is that the animation of multiple legs, wings and antennae is difficult, time-consuming and expensive. Interestingly, many music videos featured animated insects, with some of the subjectively best being Tool’s “Prison Sex” and “Vicarious”, Alice In Chains’ “I Stay Away”, and The Uncluded’s “Organs”. It seems likely that innovations in animation technology, such as CGI, have made the animation of insects easier, such that they are more readily featured. On the other hand, the time-consuming claymation and stop-motion techniques are featured in a number of these videos, including the Psychedelic Porn Crumpets’ “Tally-Ho” and They Might Be Giants’ “Insect Hospital”. It may be easier to animate insect behaviors than to induce live insects to perform. Nonetheless, live insects are featured in many videos as well.

Many such insects were readily identifiable to genus, species and sex upon examination, even to those with limited taxonomic skills. These cases included some common in the pet trade or in culture that would be readily available to insect wranglers hired to supply specimens, including the monarch (*Danaus plexippus*), the Madagascar hissing cockroach (*Gromphadorhina portentosa*), the mealworm (*Tenebrio molitor*), and the Emperor scorpion (*Pandinus imperator*). The Asian giant hornet (*Vespa mandarinia*), the honeybee (*Apis mellifera*), and the Atlas moth (*Attacus atlas*) were also noted. Odonates are generally distinct and readily identifiable from photographs [15], thus the following were noted from music videos: the Halloween pennant (*Celithemis eponina*), the widow skimmer female (*Libellula luctuosa*), the roseate skimmer (*Orthemis ferruginea*), the blue dasher (*Pachydiplax longipennis*), the twelve-spotted skimmer (*Libellula pulchella*), the flame skimmer (*Libellula saturata*), the eastern amberwing (*Perithemis tenera*), the eastern pondhawk female (*Erythemis simplicicollis*), and the ebony jewelwing (*Calopteryx maculata*). Identifiable to genus were *Atta, Catocala* and *Magicicada*. Hence, actual insects can also be used to great effect in video, although in many cases they were represented by static images or stock footage.

Music videos with insects spanned a diverse range of musical genres, including alternative, classic rock, dance, death core, folk, hard rock/metal, hip hop, new age, new wave, nu metal, pop, punk, ska, and probably others that I was unable to recognize. Apparently, insects appeal to many varieties of musicians.

### 4.3. Swarms, Chimerae and Giants

Swarms and giant insects are common in movies and are used to induce horror [11]. It is not surprising that these trends carry over into the music video genre, which have a limited amount of time in which to get the viewer’s attention. On the other hand, human–insect chimerae were often depicted in a friendly fashion. People in insect costumes or animated characters with insect wings populated quite a few videos, conveying a fun atmosphere and the fantasy of human-powered flight. This pattern is in curious opposition to that of early film, where the arthropodization of humans was common and horrific [11], and animated films, where anthropomorphic insects are used for moral and metaphor [14].

Curiously, there are few cases of insects playing instruments. Mostly these consist of musicians dressed in insect costumes. The exception was The Uncluded’s “Organs”, which opens with a grasshopper and cicada playing instruments. These are quite sensible choices, as both are sound-producing insects. In Primus’s “Shake Hands with Beef”, band members fly with insect wings, and one flies into a bug zapper at the end.

On the other hand, entomological techniques appeared in at least 11 videos. Most such videos demonstrated various forms of insect control. Dori Montana feat. Tbeezi’s “Kill The Roach” demonstrates spraying RAID insecticide and simulated stomping of insects. Korn’s “Somebody Someone” shows a variety of arthropods, and all but the fleas are squished in the end. In Mystikal’s “Tarantula”, the main character drives an exterminator truck and uses a backpack sprayer.

Not all such videos were so negative, however. Some made reference to insect collecting. The Stone Temple Pilots’ “Vasoline” shows a cricket and flies stuck to fly paper at the start, but also features a woman with an insect net and collection boxes. Marilyn Manson’s “Tourniquet” shows pinned insects, as does Rhydian’s “Parade”, which also shows many riker mounts on a wall. Others demonstrated apicultural themes. Mark Stanley’s “You Will Be Digitized” shows honeybees on a frame at the beginning. Sunna’s “Power Struggle”, Soundgarden’s “Black Hole Sun”, and The Head and the Heart’s “Honeybee” show a man in a bee suit. The Artichoke’s “The Commune” describes temporal polyethism in bees and features a lot of footage of hive activity.

Some videos took advantage of the tremendous diversity of insects. The most diversity demonstrated within a single video was Darth Nater’s “The Insect Song” and Boxcar’s “Insect (12-inch version)”, each with 10 orders. Weezer’s “All My Friends are Insects” featured seven orders, while Will Ragano’s “Cicada song” and Isaac Dunbar “Isaac’s Insects” showed five. The most diversity within an order is probably illustrated by Jesse Lane’s “Dragonfly Song”, which features mostly rotating photos of various dragonflies and a couple of damselflies (also an eclosing mayfly). Hence, the diversity of insects may be exploited artistically in the music video format, particularly with aesthetically pleasing taxa such as Odonata.

### 4.4. Date

**Figure 4:**
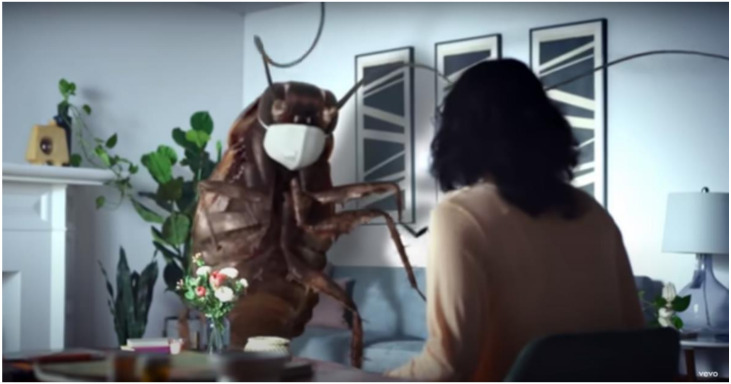
A giant, mask-wearing cockroach appears in The Offspring’s “Let the Bad Times Roll” video. ^©^ 2021 Wabi Sabi Worldwide, LLC.

The dates attributed to the songs definitely demonstrate a trend toward increasing frequency over time. It is difficult to say whether insects are being used in music videos more often because I am unable to control for growth in the output of music videos. Nonetheless, insect videos are more common. Even the older videos are attributable to artifacts. For example, the Deftones re-issued “Change (in the House of Flies)” 20 years apart (curiously, though both videos feature insects, neither one has flies.) Queen issued the video for “All Dead, All Dead” 40 years after its 1977 debut. Other older videos, such as Chubby Checker’s “The Fly”, tend to consist of footage from appearances on early TV shows. The Offspring’s “Let the Bad Times Roll” is the most recent (2021) original video, and in an homage to the current pandemic among humans, features a giant cockroach wearing a mask (Figure 4). In any case, the data should, at least, reflect current perceptions of and attitudes toward insects.

### 4.5. Best Viewed

A great many music videos featured only a brief glimpse of an insect or perhaps a band’s insect-themed logo. However, others used insects as the central character or theme that was displayed throughout nearly the entire video. These types are perhaps the most interesting for an entomologist to watch and include Alice in Chains’ “I Stay Away”, Andrew Bird’s “Imitosis”, Blind Melon’s “No Rain”, Bobby Jimmy & the Critters’ “Roaches”, Gnarls Barkley’s “Gone Daddy Gone”, The Orbweavers’ “Poison Garden”, Psychedelic Porn Crumpets’ “Tally-Ho”, PUSA’s “Ladybug”, Rammstein’s “Links 2 3 4”, S.P. Mullen & the Feel’s “Mayfly”, Southern Culture on the Skids’ “Cicada Rock 2020 (Brood IX)”, They Might Be Giants’ “Insect Hospital” and Yeah Yeah Yeahs’ “Mosquito”.

Several videos did not feature insects so prominently throughout the length of the video, but the insect was used to very dramatic effect via a brief, intimate encounter. Die Antwoord’s “Fatty Boom Boom” shows a large orthopteran (apparently a camel cricket) being birthed from a Lady Gaga look-alike. This South African hip-hop group’s stated purpose is to shock [16], and they use arthropods as a means to this end in five videos. Tyler the Creator’s “Yonkers” shows live footage of the rapper handling, eating, then vomiting up a Madagascar hissing cockroach, all in one uninterrupted take comprising the first minute of the video. Entomophagy is known to get the viewer’s attention [11], but careful examination reveals likely sleight of hand in this case. The moment before Tyler eats the roach, it goes out of frame in his left hand, then he eats something not quite identifiable from his right hand, strongly suggesting that he did not actually eat the roach. In this way, he gets the shock value of the scene without actually enduring the distaste and discomfort. In Soundgarden’s surreal “Black Hole Sun” video, one of the characters captures a lepidopteran with her elongated, animated tongue. This video also features swarms of honeybees and sphingid moths, as well as a cockroach being burned with a magnifying glass. The video is perhaps too bizarre for interpretation, though a certain degree of horror is certainly conveyed, as in the end people are drawn by gravity into the titular star to their presumed death.

### 4.6. Success

Insect videos have not been insignificant or ignored; some have won considerable honors and had presumably great impacts. MTV presented its first Video Music Awards in 1984, when “You Might Think” by The Cars won video of the year [17]. In this classic, singer Ric Ocasek’s head appears on a fly. Three other videos featuring insects have since won the award, for a success rate of >10%, a fairly remarkable achievement. Furthermore, “Virtual Insanity” by Jamiroquai won best cinematography while featuring a Madagascar hissing cockroach. The best visual effects award was won in 1990 by Tears for Fears’ “Sowing the Seeds of Love”, which shows a butterfly taking flight in the beginning. Although no insect-related music videos have won a GRAMMY award to my knowledge, Gnarls Barkley’s “Gone Daddy Gone” was nominated in 2008 [18]. 

The songs upon which the videos are based have themselves included some very popular ones. It is difficult to pick a single most successful song from this group, as there are many different charts representing different musical genres [19]. The ten songs identified will doubtless include some familiar to most readers. These records indicate that insect representation is not a barrier to success, and may in fact contribute to the success of a song and its accompanying video.

## 5. Conclusions

Music videos revealed both positive and negative representations of insects. Far from being “more feared than revered” [2], the popularity of insects in award-winning videos and high-selling songs implies that favorable views of insects may be gaining traction in western culture, as music videos are a recently developed medium. Growing coverage of insect population declines [7] and pollinators in peril [20] may be contributing to an increased awareness of insects and other arthropods. These patterns suggest that, with appropriate nudges, the attitudes of the majority can be shifted to a more positive view. Entomologists can facilitate this movement with education and outreach, such as those suggested by Duffus et al. [21] For example, teacher candidates exposed to cockroach projects enjoy greatly reduced fear and disgust toward them [22]. By taking opportunities to present insects to popular audiences, using social media to counteract insect myths and promote factual phenomena or interesting new breakthroughs in insect science, we can help move popular perception in a favorable direction.

## Figures and Tables

**Table 1:** The grouping of arthropod taxa observed in the study.

Taxon	Arthropods Observed
Araneae	spider, tarantula
Blattodea	cockroach
Coleoptera	beetle, weevil
Diptera	cranefly, fly, maggot, mosquito
Ephemeroptera	mayfly
Homoptera	cicada
Hymenoptera	ant, bee, wasp
Lepidoptera	butterfly, caterpillar, moth, pupa
Mantodea	mantis
Myriapoda	centipede, millipede
Neuroptera	dobsonfly, lacewing
Odonata	damselfly, dragonfly
Orthoptera	cricket, grasshopper, katydid
Other Arachnida	harvestman, scorpion, tick, vinegaroon, whipscorpion
Phasmida	stick insect, leaf insect
Psocodea	louse
Siphonaptera	flea
Trichoptera	caddisfly
Unknown	unidentifiable or unclassifiable

**Table 2:** Insect-related music videos winning the MTV Video Music Award.

Artist	Title	Year
The Cars	You Might Think	1984
Jamiroquai	Virtual Insanity	1997
Missy Elliott	Work It	2003
Taylor Swift	You Need to Calm Down	2009

**Table 3:** Chart-topping songs having music videos featuring insects.

Artist	Song Title	Charts
Blind Melon	No Rain	mainstream rock, alternative
Godsmack	I Stand Alone	mainstream rock
Katie Perry	Wide Awake	mainstream top 40, adult top 40, dance club
Linkin Park	What I’ve Done	mainstream rock, rock and metal, alternative
No Doubt	Don’t Speak	hot 100 airplay
Soundgarden	Black Hole Sun	alternative
Stone Temple Pilots	Vasoline	mainstream rock
Talk Talk	It’s My Life	US hot dance club
Tears For Fears	Sowing the Seeds of Love	Cash Box Top 100
The Cars	You Might Think	mainstream rock

## Supplementary Materials

The following are available online at https://www.mdpi.com/article/10.3390/insects12070616/s1, File S1, Raw data.
